# Myelomonocytic skewing in chronic myelomonocytic leukemia: phenotypic, molecular and biologic features and impact on survival

**DOI:** 10.1111/ejh.13577

**Published:** 2021-03-08

**Authors:** Klaus Geissler, Eva Jäger, Agnes Barna, Temeida Graf, Elmir Graf, Leopold Öhler, Gregor Hoermann, Peter Valent

**Affiliations:** ^1^ Medical School Sigmund Freud University Vienna Austria; ^2^ Department of Internal Medicine V with Hematology, Oncology and Palliative Medicine Hospital Hietzing Vienna Austria; ^3^ Department of Laboratory Medicine Medical University of Vienna Vienna Austria; ^4^ Blood Transfusion Service Blood Transfusion Service for Upper Austria Austrian Red Cross Linz Austria; ^5^ Department of Internal Medicine/Oncology St. Josef Hospital Vienna Austria; ^6^ Central Institute of Medical and Chemical Laboratory Diagnostics Medical University of Innsbruck Innsbruck Austria; ^7^ Ludwig Boltzmann Institute for Hematology and Oncology (LBI HO) Medical University of Vienna Vienna Austria; ^8^ Department of Internal Medicine I Division of Hematology and Hemostaseology Medical University of Vienna Vienna Austria

**Keywords:** CMML, in vitro cultures, NGS, prognosis, skewing

## Abstract

**Background:**

Myelomonocytic skewing is considered as a key pathophysiologic phenomenon in chronic myelomonocytic leukemia (CMML), but its prevalence and potential correlation with phenotypic, genotypic, and clinical features are poorly defined.

**Methods:**

Skewed differentiation toward the myelomonocytic over erythroid commitment as indicated by an inverse ratio of myelomonocytic/erythroid colonies was investigated in 146 patients with CMML by semisolid *in vitro* cultures.

**Results:**

There was a high prevalence of myelomonocytic skewing in patients with CMML (120/146, 82%); whereas, this phenomenon was rare in normal individuals (1/98, 1%). Patients with CMML with myelomonocytic skewing had higher white blood cell and peripheral blast cell counts, and lower platelet values. The number of mutations in genes of the epigenetic and/or splicing category was higher in CMML patients with as compared with patients without skewing. Patients with myelomonocytic skewing had more frequently mutations in RASopathy genes and higher growth factor independent myeloid colony formation. Interestingly, the lack of myelomonocytic skewing discriminated patients with CMML with a particularly favorable prognosis (60 vs 19 months, *P* = .003) and a minimal risk of transformation.

**Conclusion:**

Myelomonocytic skewing as determined by semisolid cultures can discriminate subgroups of patients with CMML with a different phenotype, a different genotype, and a different prognosis.

**Novelty statement**

- Myelomonocytic skewing as determined by semisolid in vitro cultures has been performed in a large cohort of patients with chronic myelomonocytic leukemia (CMML).
- Myelomonocytic skewing can discriminate subgroups of patients with CMML with a different phenotype, a different genotype and a different prognosis.
- Our findings may be important for the understanding and management of CMML.

## 1 INTRODUCTION

Normal hematopoietic function is maintained by a well controlled balance of myelomonocytic, mega erythroid, and lymphoid progenitor cell populations. This balance may be skewed during pathologic conditions such as hematological malignancies, infections, and autoimmunity but also in aged hematopoiesis.^1^, ^2^, ^3^, ^4^, ^5^, ^6^, ^7^

Recently, we have reported that analysis of myelomonocytic skewing *in vitro* may be useful to investigate skewed differentiation toward the myelomonocytic over erythroid commitment in patients.^8^, ^9^ Since the presence of skewing may be associated with a different phenotype, a different mutational landscape and a different prognosis in patients with myeloid malignancies this *in vitro* test may help to comprehensively study hematopoiesis in patients with complex disturbances of blood formation. Myelomonocytic skewing has been reported in chronic myelomonocytic leukemia (CMML) by analyzing single‐cell‐derived colonies, but this phenomenon has not been correlated with phenotype and clinical characteristics.^10^ The aim of this study was to study the prevalence and a potential correlation of myelomonocytic skewing as determined by semisolid *in vitro* cultures with phenotypic, molecular, biologic, and clinical features in a large cohort of patients with CMML.

## 2 METHODS

### 2.1 Patients

In the “Austrian Biodatabase for Chronic Myelomonocytic Leukemia” (ABCMML) clinico‐laboratory, real‐life data have been captured from 606 patients with CMML from 14 different hospitals over the last 30 years. The ABCMML has been shown to be a representative and useful real‐life data source for further biomedical research.^11^ In 146 patients with CMML of our ABCMML data from semisolid *in vitro* cultures were available which were used for this retrospective study. This research has been approved by the ethic committee of the City of Vienna on 10.06.2015 (ethic code: 15‐059‐VK).

### 2.2 Colony Assay

In one of our centers (Medical University of Vienna), the assessment of hematopoietic colony formation *in vitro* has been an integral part of the diagnostic work up in patients with suspected myeloid malignancies for many years.^12^ Colony‐forming unit‐ granulocyte/macrophage (CFU‐GM) and burst‐forming unit‐erythroid (BFU‐E) growth were assessed in semisolid cultures with and without growth factors as previously described.^13^, ^14^ Mononuclear cells (MNC) were isolated from peripheral blood (PB) of patients by Ficoll‐Hypaque density gradient centrifugation (density 1.077 g/mL, 400 *g* for 40 minutes). The low‐density cells were collected from the interface between density solution and plasma, washed twice, and resuspended in Iscove‘s modified Dulbecco‘s medium (GIBCO, Paisley, Scotland). PBMNCs were cultured in 0.9% methylcellulose, 30% fetal calf serum (FCS; Biomedica, Vienna, Austria), 10% bovine serum albumin (Sigma), α‐thioglycerol (10^‐4^ mol/L), and Iscove‘s modified Dulbecco‘s medium. For stimulation of progenitor cells, cultures were supplemented with recombinant human granulocyte‐macrophage colony‐stimulating factor (GM‐CSF) (10 ng/mL; R&D Systems), rh‐interleukin‐3 (10 ng/mL; R&D Systems) and erythropoietin (EPO, 2 U/mL; Roche). Stimulated cultures were plated in duplicates at 100 × 10^3^ PBMNC/mL. Unstimulated cultures were plated in duplicates or triplicates, respectively, at 25‐100 × 10^3^ PBMNC/mL. Plates were incubated at 37°C, 5% CO2, and full humidity. After a culture period of 14 days, cultures were examined under an inverted microscope. Aggregates with more than 40 translucent, dispersed cells were counted as CFU‐GM. Bursts containing more than 100 red‐colored cells were scored as BFU‐E. Progenitor cell data are expressed as mean values from cultures. In general, progenitor cell cultures were performed at diagnosis and prior any cytoreductive treatment.

### 2.3 Molecular studies

Genomic DNA was isolated from mononuclear cell (MNC) fractions of these blood samples according to standard procedures. The mutational status of CMML‐related protein coding genes was determined by targeted amplicon sequencing using the MiSeq platform (Illumina). Details regarding gene panel, library preparation, and data processing have been reported previously.^11^ Only variants with an allelic frequency (VAF) ≥5%, a described population frequency (MAF) <1%, and an annotated pathogenic effect (or probability >90% of being pathogenic) were included, with pathogenicity determined according to databases as shown in Table S1 and published studies.

### 2.4 Statistical analysis

The log‐rank test was used to determine whether individual parameters were associated with OS. OS was defined as the time from sampling to death (uncensored) or last follow‐up (censored). Multivariate Cox regression analysis of overall survival was used to describe the relation between the event incidence, as expressed by the hazard function and a set of covariates. Dichotomous variables were compared between different groups with the use of the chi‐square test. The Mann‐Whitney *U* test was used to compare two unmatched groups when continuous variables were not normally distributed. Results were considered significant at *P* < .05. Statistical analyses were performed with the SPSS version 19.0.0 (SPSS Inc); the reported *P* values were 2‐sided.

## 3 RESULTS

There was a high prevalence of myelomonocytic skewing as indicated by an inverse ratio of CFU‐GM/BFU‐E in patients with CMML (120/146, 82%); whereas, this phenomenon was rare in normal individuals (1/98, 1%). As shown in Table 1, there was no difference in patients with and without myelomonocytic skewing with regard to age and male predominance.

**TABLE 1:** Phenotype of patients with CMML stratified by the presence or absence of myelomonocytic skewing

Variables	All patients with CMML (n=146)	CMML patients with skewing (n=120, 82%)	CMML patients without skewing (n=26, 18%)	*P* value
Age; median (range)	72.5 (36‐92)	72 (45‐92)	73 (36‐92)	.529
Sex (Male); n (%)	84 (58)	70 (58)	14 (54)	.675
WBC G/L, median (range)	15.5 (2.8‐156)	17.7 (2.8‐156)	8.3 (3.1‐38)	<.001
Hb g/dL, median (range)	11.1 (4.3‐15)	11.0 (4.3‐15)	12.0 (8.2‐14.8)	.051
PLT G/L, median (range)	115 (5.867)	100 (5‐867)	160 (35‐689)	.002
Blasts %, median (range)	0 (0‐17)	0 (0‐17)	0 (0‐2)	.018
Splenomegaly n (%)	32/105 (30%)	28/86 (33%)	4/18 (22%)	.390

### 3.1 Impact of myelomonocytic skewing on the phenotype of CMML

The phenotype stratified by the presence or absence of myelomonocytic skewing in patients is shown in Table 1. Patients with CMML with myelomonocytic skewing had higher white blood cell (WBC) and PB blast cell counts, a trend toward lower hemoglobin (Hb) values and significantly lower platelet (PLT) counts as compared with patients without skewing. The incidence of splenomegaly was not significantly different.

### 3.2 Impact of myelomonocytic skewing on survival and time to AML transformation of CMML patients

**FIGURE 1:**
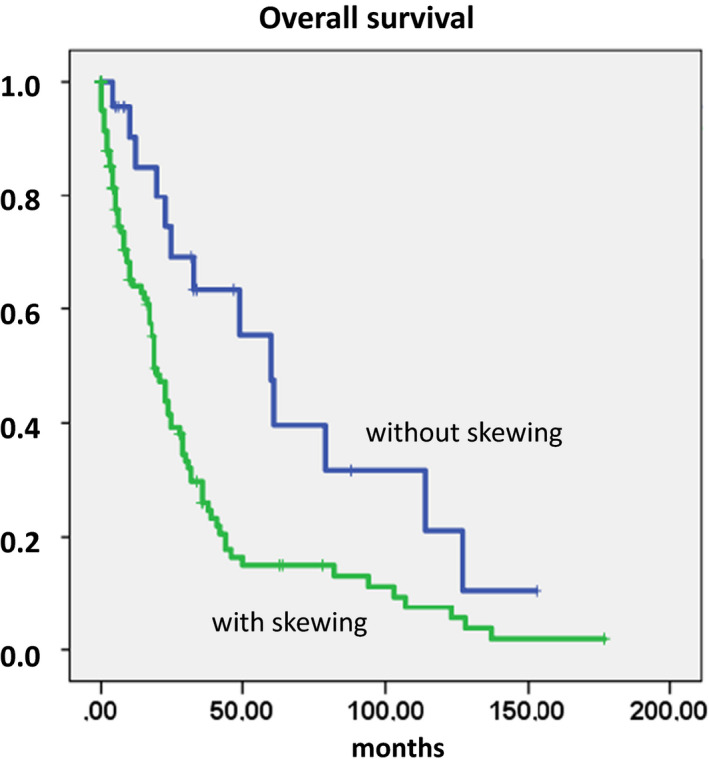
Overall survival in patients with CMML stratified by the presence or absence of myelomonocytic skewing

Figure 1 shows the Kaplan‐Meier plots of overall survival in patients with CMML stratified by the presence or absence of myelomonocytic skewing. Interestingly, the lack of myelomonocytic skewing discriminated patients with CMML with a particularly favorable prognosis. The median survival of patients with CMML with myelomonocytic skewing was 19 months as compared with 60 months in patients without skewing (*P* = .003). In Figure S1, the Kaplan‐Meier plots of other established single prognostic factors such WBC count, Hb value, PLT count, and PB blasts are shown. All these parameters also had a prognostic impact in our study. In order to determine the relation of the prognostic impact of myelomonocytic skewing to other established prognostic factors, several Cox regression analyses were performed adjusting for these factors. As shown in Table S2, myelomonocytic skewing, not unexpectedly, lost its prognostic significance if adjusted for WBC, but retained its significance in the presence of all other parameters.

**FIGURE 2:**
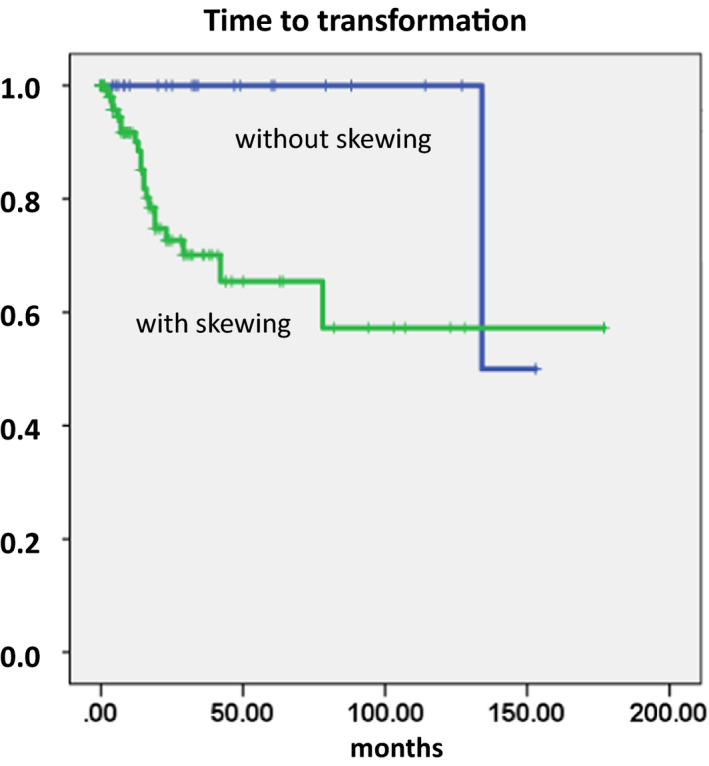
Time to AML transformation in patients with CMML stratified by the presence or absence of myelomonocytic skewing

Figure 2 shows the time to AML transformation stratified by the presence or absence of myelomonocytic skewing. Lack of myelomonocytic skewing discriminated patients with CMML with a minimal risk of transformation (*P* = .012). In fact, these patients had at 10 years 0% risk of transformation into AML as compared with 43% in patients with skewing. There was one patient who had no myelomonocytic skewing at the time of diagnosis and developed AML after 134 months. In Figure S2, the Kaplan‐Meier plots for time to transformation is given for WBC count, Hb value, PLT count, and PB blasts. Except for the PB blast cell counts, none of these parameters had a significant impact. As shown in Table S3, myelomonocytic skewing retained its significance in the presence of all these parameters.

In a subgroup of patients, cytogenetic and/or molecular information was available (n = 82). The Kaplan‐Meier plots of time to AML transformation in patients with CMML stratified by the presence or absence of genetic variables which were defined as high risk aberrations in the CPSS‐Mol Score are shown in Figure S3.^15^ There was a trend toward a significant difference (*P* = .114), but this may be due to the limited number of patients with genetic information.

### 3.3 Mutational profile of CMML stratified by the presence or absence of myelomonocytic skewing

Mutations in genes of the epigenetic control and the splicing machinery have shown to promote differentiation toward the myelomonocytic cell lineage in preclinical mouse models.^1^, ^2^, ^3^ Therefore, we determined the proportion of patients with mutations in both gene categories as well as the median number of mutations of both categories in patients with CMML with and without skewing as determined by semisolid *in vitro* cultures. As shown in Table 2, there was no difference in the proportion of patients with mutations in genes of the epigenetic and/or splicing category but a higher number of mutations in CMML patients with as compared with patients without skewing (md number 2 vs 1). Moreover, patients with myelomonocytic skewing had more frequently mutations in RASopathy genes as compared with patients without skewing (58% vs 25%).

**TABLE 2:** Molecular aberrations in patients with CMML patients stratified by the presence or absence of myelomonocytic skewing

Molecular Variables	All patients with CMML (n=82)	CMML patients with skewing (n=66)	CMML patients without skewing (n=16)	P Value
Patients with mutations in the epigenetic gene category, n (%)	75 (91%)	62 (94%)	13 (81%)	.103
Patients with mutations in the splicing gene category, n (%)	43 (52%)	37 (56%)	6 (38%)	.182
Patients with mutations in the epigenetic and/or splicing category, n (%)	42 (51%)	64 (97%)	15 (94%)	.538
Mutation numbers in the epigenetic and/or splicing category, median (range)	2 (0‐4)	2 (0‐4)	1 (0‐4)	.016
Patients with mutations in RASopathy genes, n (%)	32 (39)	38 (58)	4 (25)	.019

Genes analyzed by NGS—epigenetic category: TET2, DNMT3A, ASXL1, EZH2, and IDH 1 and 2; splicing category: SRSF2, SF3B1, U2AF1, and ZRSR2; and RAS category: NRAS, KRAS, CBL, NF1, and PTPN11

### 3.4 Impact of myelomonocytic skewing on spontaneous myeloid colony formation

**FIGURE 3:**
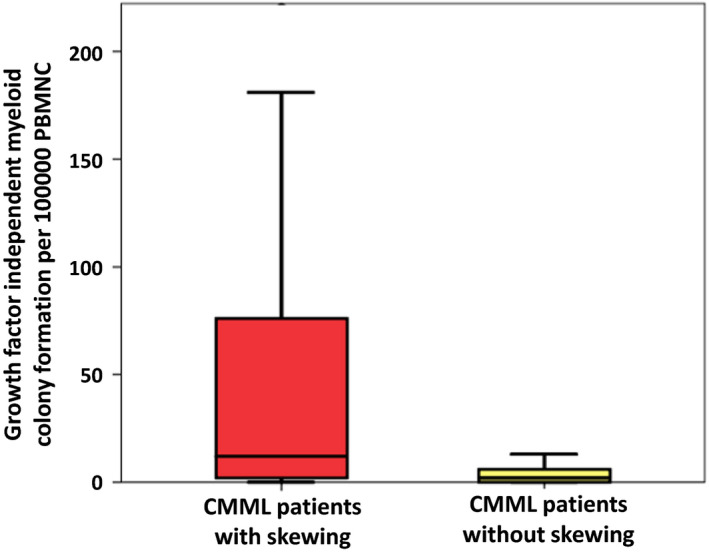
Box plots showing the distribution of spontaneous myeloid colony numbers in patients with CMML stratified by the presence or absence of myelomonocytic skewing including median values, minimum values, maximum values, as well as upper and lower quartiles, respectively. Cultures were plated in duplicates or triplicates, respectively, at 25‐100 × 10^3^ PBMNC/mL. Aggregates with more than 40 translucent, dispersed cells were counted as CFU‐GM. CFU‐GM data from patients are expressed as mean values from cultures.

*In vitro* cultures data were available from 146 patients. We recently were able to show that growth factor independent CFU‐GM formation is a functional surrogate of RAS‐pathway activation.^16^, ^17^ The spontaneous formation of CFU‐GM in normal individuals (median 4.8/10^5^ PBMNC, range 3.5‐8.5) has been reported by us previously.^18^ The numbers of spontaneously formed CFU‐GM in patients with CMML stratified by the presence or absence of myelomonocytic skewing is indicated in Figure 3. The box plots show a large variation in colony numbers between single patients in the two cohorts; however, median CFU‐GM numbers per 10^5^ MNC were significantly higher in patients with myelomonocytic skewing (md 11, range 0‐1127, n = 107) as compared with patients without skewing (md 2, range 0‐167, n = 22; *P* = .0067).

### 3.5 Temporal relationship of myelomonocytic skewing and RAS‐pathway activation in a CMML patient with serial in vitro cultures

In one CMML patient, serial *in vitro* cultures were performed during the course of disease. As shown in Table 3, the transition to myelomonocytic skewing (from a CFU‐GM/BFU‐E ratio <1 to >1) was accompanied by an increase in the number of unstimulated CFU‐GM. This patient had a WBC count of 8.5 G/L in 3/11 indicating MDS‐CMML and later progressed to MPN‐CMML in 2/16 with a WBC count of 16.6 G/L. Moreover, NGS analysis revealed the emergence of an NRAS clone at the same time. Thus, we can demonstrate hyperactivation of the RAS signaling pathway in this patient at the molecular and functional level.

**TABLE 3:** Stimulated and unstimulated colony formation in a CMML patient with serial *in vitro* cultures

	Stimulated CFU‐GM	Stimulated BFU/E	CFU‐GM/ BFU‐E ratio	Unstimulated CFU‐GM	NRAS VAF
Sample 3/11	28	55	0.51	8	<5%
Sample 2/16	398	262	1.52	170	19%
Sample 8/16	654	169	3.87	381	41%
Controls (n=80)	9 (1‐44)	33 (5‐91)	0.3 (0.1‐1.2)	4.5 (0‐8.5)	<5%

## 4 DISCUSSION

CMML is a hematopoietic malignancy of the elderly that is characterized by overlapping features of myelodysplastic syndromes (MDS) and myeloproliferative neoplasms (MPN) and an inherent risk of transformation to secondary acute myeloid leukemia. Several articles extensively reviewed the diagnostic criteria, and the clinical and molecular characteristics of CMML, but the biological features of this disease are not comprehensively reported.^19^, ^20^, ^21^, ^22^, ^23^ We have originally shown the *in vitro* characteristics of CMML in a small number of patients.^24^ In this study, we performed cell‐culture studies in four patients with CMML and demonstrated the following *in vitro* features: excessively increased circulating myelomonocytic progenitor cells, while erythroid progenitor cells were either moderately increased or not detectable indicating a shift of hematopoiesis toward the myelomonocytic lineage. Moreover, growth factor independent myeloid colony formation was observed in a subgroup of patients.

Myelomonocytic skewing has been proposed as a key phenomenon in the pathobiology of CMML. In a seminal paper using mutation‐specific discrimination analysis of single‐cell‐derived colonies in 28 patients with CMML, Itzykson et al could show that the main characteristics of this disease are early clonal dominance, arising at the CD34+/CD34‐stage of hematopoiesis, and granulomonocytic differentiation skewing of multipotent and common myeloid progenitors,^10^ but this phenomenon has not been correlated to other features such as phenotype and clinical outcome. Since semisolid *in vitro* cultures from PBMNCs of normal individuals usually contain a higher concentration of BFU‐E as compared to CFU‐GM, this test may be useful for investigating skewed differentiation toward the myelomonocytic over erythroid commitment in patients.^8^, ^9^ Due to the fact that in our center the assessment of hematopoietic colony formation *in vitro* has been an integral part of the diagnostic work up in patients with suspected myeloid malignancies for many years^12^ and these data are part of the ABCMML,^11^ we had the possibility to analyze the phenomenon of myelomonocytic skewing in a relatively large cohort of patients with CMML in this retrospective study. We show that myelomonocytic skewing as measured by our *in vitro* culture system is a common finding in patients with CMML and is associated with a different phenotype including higher WBC and PB blast cell counts and lower PLT values. The biological significance of our finding was supported by the observation that the small proportion of patients with CMML without skewing had a remarkable good prognosis and a minimal risk of AML transformation as compared with patients with skewing. Whereas, the prognostic significance of this finding was retained in the presence of established prognostic factors including Hb, PLT, and blasts in the multivariate analysis; it was lost after adjustment for WBC. This finding might indicate that myelomonocytic skewing and the development of leukocytosis in CMML may be part of the same biologic phenomenon.

In this study, we show that patients with myelomonocytic skewing had a higher number of mutations in the genes of the epigenetic and/or splicing category. This finding is in agreement with preclinical mouse models^1^, ^2^ but also with findings in myelofibrosis patients in whom the presence of myelomonocytic skewing was associated with a higher frequency of additional mutations, particularly in genes of the epigenetic and/or splicing machinery.^8^

Moreover, we demonstrate in our patients with CMML a significant association between the presence of myelomonocytic skewing and the activation of the RAS signaling pathway, both at the molecular and at the functional level. We also show in a patient with serial *in vitro* cultures that the development of myelomonocytic skewing was accompanied by the transition of MD‐CMML into MP‐CMML and by RAS‐pathway activation. Our findings do not allow to make definitive conclusions regarding the temporal relationship between these biological phenomena. Considering the much higher frequency of myelomonocytic skewing as compared with RAS‐pathway activation in our patients, however, it is more likely that myelomonocytic skewing may be the earlier event in CMML and predisposes hematopoietic cells for the subsequent development of RAS‐pathway activation. This hypothesis is supported by findings from preclinical models showing that changes in genes of the epigenetic machinery may cause skewing of myelopoiesis over erythropoiesis and changes in components of the RAS‐pathway are associated with growth‐factor independent myeloid colony formation *in vitro*.^2^, ^25^, ^26^, ^27^, ^28^

The well known unfavorable impact of leukocytosis and anemia in CMML may be considered as indirect evidence that skewing of hematopoiesis toward the myelomonocytic lineage predict inferior outcome. The prognostic impact of myelomonocytic skewing at the progenitor cell level in CMML, however, has not been shown to best of our knowledge so far. We think that myelomonocytic skewing as demonstrated by in vitro cultures may be a more robust parameter of skewed differentiation toward the myelomonocytic over erythroid commitment because the WBC count and Hb values are more easily confounded by other condition such as infection and bleeding, which usually do not change the progenitor cell compartment.^29^, ^30^

We conclude that myelomonocytic skewing can discriminate patients with CMML with a different phenotype and different prognosis. Moreover, myelomonocytic skewing seems to predispose for the emergence of additional molecular aberrations in genes of the RAS‐pathway which will finally result in MP‐CMML and transformation. Therefore, therapeutic strategies targeting the molecular mechanisms underlying the biologic phenomenon of myelomonocytic skewing may be an attractive approach to impact CMML.

## CONFLICT OF INTEREST

The authors declare no conflict of interest.

## AUTHORS’ CONTRIBUTIONS

E.J. performed colony assays; A.B. performed NGS analyses; T.G. and E.G. performed the administration of data. G.H. interpreted molecular data; L.O. and P.V. provided patient samples and clinical information; K.G. directed the research, collected, analyzed, and interpreted the data and wrote the manuscript. All authors had the opportunity to review the manuscript.

## Supporting information

Supplementary Material

## Data Availability

The data that support the findings of this study are available from the corresponding author, [KG], upon reasonable request.
