# Supplementary material for: Myelomonocytic skewing in chronic myelomonocytic leukemia: phenotypic, molecular and biologic features and impact on survival
Source: Eur J Haematol. 2021 Mar 8;106(5):627–33. doi: 10.1111/ejh.13577 (PMC8554855; doi:10.1111/ejh.13577)
Supplement: Supplementary file 1 — Supplementary Material [file EJH-106-627-s001.docx]

Supplementary Information

**Figure S1**

**Figure S2**

**Figure S3**

**Table S1:** Databases and respective links used for determination of pathogenicity of variants

| **Database** | **Link** |
| --- | --- |
| ClinVar | [www.ncbi.nlm.nih.gov/clinvar/](https://www.ncbi.nlm.nih.gov/clinvar/) |
| COSMIC | <https://cancer.sanger.ac.uk/cosmic> |
| ENSEMBL | <https://www.ensembl.org/index.html> |
| PolyPhen | <http://genetics.bwh.harvard.edu/pph2/> |
| MutationTaster | <http://www.mutationtaster.org/> |
| SIFT | <https://sift.bii.a-star.edu.sg/> |
| gnomAD | <http://gnomad.broadinstitute.org/> |
| ESP | [http://evs.gs.washington.edu/EVS/](http://evs.gs.washington.edu/EVS/ ) |
| ExAC | <http://exac.broadinstitute.org/> |
| dbNSFP | <http://sites.google.com/site/jpopgen/dbNSFP> |
| dbSNP | <https://www.ncbi.nlm.nih.gov/snp> |
| CG69 | <http://www.completegenomics.com/public-data/69-Genomes/> |
| G1000 | <https://www.internationalgenome.org/1000-genomes-browsers/> |

**Table S2:** Hazard ratios, confidence intervals and p-values of Cox regression analyses for survival including myelomonocytic skewing and established prognostic parameters

| **Parameter** | **Hazard Ratio** | **95% Confidence Interval** | **P-Value** |
| --- | --- | --- | --- |
| Skewing present | 1.76 | 0.92-3.35 | 0.085 |
| WBC >13 G/L | 1.69 | 1.06-2.69 | 0.028 |
|  | | | |
| Skewing present | 2.33 | 1.29-4.18 | 0.005 |
| Hb <10 g/dL | 1.54 | 1.004-2.38 | 0.048 |
|  | | | |
| Skewing present | 2.21 | 1.22-3.98 | 0.009 |
| PLT <100 G/L | 2.20 | 1.46-3.30 | <0.001 |
|  | | | |
| Skewing present | 2.06 | 1.13-3.74 | 0.018 |
| PB Blasts present | 2.25 | 1.45-3.48 | <0.001 |

WBC: white blood cell counts; Hb, hemoglobin; PLT, platelets; PB, peripheral blood

**Table S3:** Hazard ratios, confidence intervals and p-values of Cox regression analyses for risk of transformation including myelomonocytic skewing and peripheral blood parameters

| **Parameter** | **Hazard Ratio** | **95% Confidence Interval** | **P-Value** |
| --- | --- | --- | --- |
| Skewing present | 9.09 | 1.10-74.89 | 0.040 |
| WBC >13 G/L | 0.98 | 0.39-2.41 | 0.956 |
|  | | | |
| Skewing present | 8.92 | 1.17-68.00 | 0.035 |
| Hb <10 g/dL | 1.27 | 0.49-3.30 | 0.622 |
|  | | | |
| Skewing present | 8.75 | 1.14-66.85 | 0.037 |
| PLT <100 G/L | 1.53 | 0.64-3.67 | 0.343 |
|  | | | |
| Skewing present | 7.89 | 1.02-61.14 | 0.048 |
| PB Blasts present | 2.83 | 1.16-6.92 | 0.022 |

WBC: white blood cell counts; Hb, hemoglobin; PLT, platelets; PB, peripheral blood
